# Cardio-Oncology: Cancer Therapy-related Cardiovascular Complications in a Molecular Targeted Era: New Concepts and Perspectives

**DOI:** 10.7759/cureus.1258

**Published:** 2017-05-18

**Authors:** David Hurtado-de-Mendoza, Arturo Loaiza-Bonilla, Paula A Bonilla-Reyes, Gabriel Tinoco, Rodrigo Alcorta

**Affiliations:** 1 University of Miami Miller School of Medicine, University of Miami Miller School of Medicine/Jackson Memorial Hospital, Florida, USA; 2 Medicine, Hematology and Oncology, Cancer Treatment Centers of America; 3 Facultad de Medicina, Pontificia Universidad Javeriana; 4 Department of Internal Medicine, The Ohio State University College of Medicine; 5 Facultad de Medicina, Universidad Peruana Cayetano Heredia

**Keywords:** cancer treatment, cardiac dysfunction, cardiotoxicity, cardio-oncology, cardiovascular events

## Abstract

Cardio-oncology is a medical discipline that identifies, prevents, and treats the cardiovascular complications related to cancer therapy. Due to the remarkable proliferation of new cancer therapies causing cardiovascular complications, such as hypertension, heart failure, vascular complications, and cardiac arrhythmia, we provide an extensive, comprehensive revision of the most up-to-date scientific information available on the cardiovascular complications associated with the use of newer, novel chemotherapeutic agents, including their reported incidence, suggested pathophysiology, clinical manifestations, potential treatment, and prevention. The authors consider this topic to be relevant for the clinicians since cardiovascular complications associated with the administration of recently approved drugs are relatively underappreciated.

The purpose of this article is to provide a state-of-the-art review of cardiovascular complications associated with the use of newer, novel chemotherapeutic agents and targeted therapies, including their reported incidence, suggested pathophysiology, clinical manifestations, potential treatment, and prevention.

Ongoing efforts are needed to provide a better understanding of the frequency, mechanisms of disease, prevention, and treatment of cardiovascular complications induced by the newer, novel chemotherapeutic agents. Development of a cardio-oncology discipline is warranted in order to promote task forces aimed at the creation of oncology patient-centered guidelines for the detection, prevention, and treatment of potential cardiovascular side effects associated with newer cancer therapies.

## Introduction and background

Cancer is one of the top leading causes of death in the world. As a result of improved survival with novel cancer therapies, cardiovascular disease is a prominent cause of death in many cancer survivors, with cardiotoxicity being a serious side effect of chemotherapy and radiation therapy. The cardiotoxicity profile of the various chemotherapeutic agents, mechanisms of disease and potential approaches to prevention of cardiovascular disease differ substantially. While the cardiotoxic effects of time-honored chemotherapeutic agents, such as anthracyclines and alkylating agents, are well recognized and extensively studied, the cardiovascular complications associated with the administration of recently approved drugs are relatively underappreciated.

The purpose of this article is to provide a state-of-the-art review of cardiovascular complications (i.e., hypertension, myocardial ischemia and infarction (MI), heart failure, thromboembolism, QT prolongation, and arrhythmias) associated with the use of newer, novel chemotherapeutic agents and targeted therapies, including their reported incidence, suggested pathophysiology, clinical manifestations, potential treatment, and prevention. 

## Review

### Small molecule tyrosine kinase inhibitors

The human genome contains about 90 tyrosine kinase and 43 tyrosine kinase (TK)-like genes whose expression translates into two important groups: transmembrane receptor and intracellular non-receptor tyrosine kinases. The overexpression and/or mutation of tyrosine kinase signaling proteins have been shown to cause abnormal cell proliferation and differentiation, angiogenesis, and inhibition of apoptosis [1-2].

**Figure 1:**
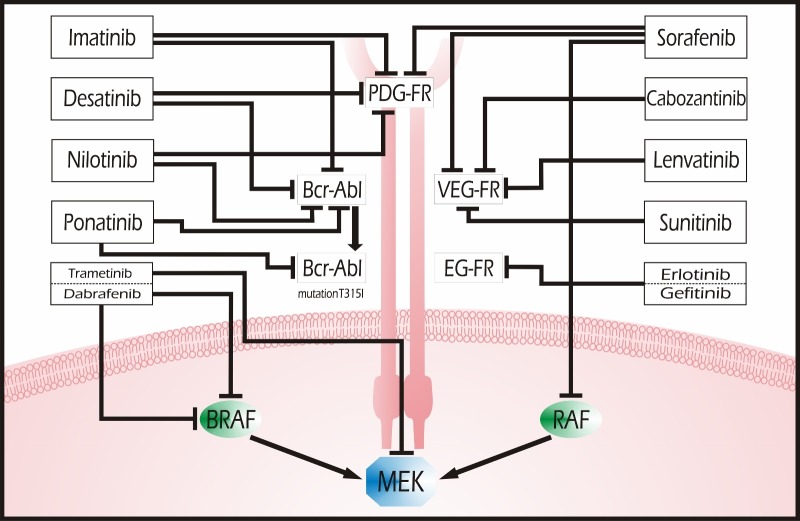
Small Molecule Tyrosine Kinase Inhibitors The overexpression and/or mutation of tyrosine kinase signaling proteins has been shown to cause abnormal cell proliferation and differentiation, angiogenesis, and inhibition of apoptosis. Tyrosine kinase inhibitors (TKIs) are small molecules that inhibit phosphorylation and, hence, activation of kinases by targeting them at the receptor or intracellular level. Since tyrosine kinases are ubiquitous in distribution, TKIs can adversely affect multiple organs, including the heart. Figure 1 shows the activity of each inhibitor drug on the different kinases.

Tyrosine kinase inhibitors (TKIs) are small molecules that inhibit phosphorylation, and hence activation, of tyrosine kinases [3]. The discovery that administration of imatinib mesylate (i.e., Gleevec®), a TKI, dramatically improved survival in patients with chronic myeloid leukemia (CML) rapidly advanced the development and application of molecular-targeted therapies [4]. Since tyrosine kinases are ubiquitous in distribution, TKIs can adversely affect multiple organs, including the heart [5]. Figure 1 summarizes the main targets of these agents as well as the common mechanisms. 

Imatinib Mesylate

Imatinib mesylate targets multiple tyrosine kinases, including Bcr-Abl (the fusion protein encoded by the Philadelphia chromosome), c-Kit (the stem cell factor receptor), and platelet-derived growth factor receptor (PDGFR)-α and β. It is the drug of choice for the treatment of CML and indicated as the first-line or adjuvant therapy for individuals with Philadelphia chromosome-positive pre-B cell acute lymphoblastic leukemia (B-ALL), gastrointestinal stromal tumors (GIST), and acute and/or chronic eosinophilic leukemia (CEL).

Of patients treated with imatinib monotherapy, 0.5% to 1.7% develop heart failure symptoms due to left ventricular (LV) systolic dysfunction [6]. Initial studies that used serum B-type natriuretic peptide or troponin T levels as a marker of cardiac function reported no cardiotoxicity with imatinib therapy [7]; however, noninvasive imaging studies have demonstrated a decline in the left ventricular ejection fraction with therapy [8]. Although pathological findings characteristic of toxin-induced myopathy have been demonstrated on biopsy in imatinib-treated patients [8], studies have failed to correlate pathologic findings with clinical evidence of cardiac dysfunction. Which patients are most susceptible to developing cardiac dysfunction with imatinib therapy and are candidates for appropriate preventative and management interventions is currently unknown.

Dasatinib

Currently indicated for the treatment of CML and Philadelphia chromosome (+) ALL (acute lymphoblastic leukemia) after imatinib failure, dasatinib is a very potent TKI targeting Bcr-Abl, cKit, PDGFR-α and -β, and the Src family of kinases [9]. The most commonly associated adverse cardiovascular effect is peripheral edema. Heart failure incidence is reported to range between 2% to 4% [9-10]. Dasatinib treatment is associated with asymptomatic QT interval prolongation (≥ 500 ms) in 2% to 3% of patients, and isolated cases of pericardial effusion have also been reported [6, 10]. Based on similar pharmacodynamic profiles, imatinib and dasatinib-induced cardiotoxicity likely have a common mechanism of action.

Nilotinib

Nilotinib is an inhibitor of Bcr-Abl, c-Kit, and PDGFR-α and -β receptors. Its potency in vitro is 30-fold compared to imatinib, and it is used as the second-line treatment in patients with CML initially treated with imatinib and also used in those patients with Philadelphia positive B-ALL. The major cardiac event reported is an increased incidence of QT prolongation (1% to 10% incidence), warranting the issuance of a black box warning from FDA as part of the agreement for its approval [6, 11]. An experiment performed on canine heart myocytes demonstrated that the mechanism behind this action potential delay is an inhibition of phosphoinositide-3-kinase due to “on-target” effects of nilotinib. As a result, multiple ion channels are decreased (including delayed K+ currents IKr and IKs, L-type calcium ion current ICa, L, and peak sodium ion current INa) and persistent Na+ current INaP is increased [12]. Strict monitoring and repletion of magnesium and electrolyte levels are warranted when using this agent.

Lapatinib

Lapatinib is an orally administered quinazoline targeting epidermal growth factor receptor (EGFR) and ErbB2, which is also the target of the monoclonal antibody, trastuzumab (see below), commonly associated with significant cardiotoxicity. ErbB receptors cause carcinogenesis by complexing with phosphoinositide 3-kinase (PI3K) complexes to activate the serine-threonine kinase Akt pathway [13-14].

In a large randomized trial of combination chemotherapy with lapatinib for metastatic breast cancer, 2.5% of patients had a marked decrease (> 20% decline from baseline) in left ventricle ejection fraction (LVEF) without heart failure symptoms [15]. In a pooled analysis of 3,689 patients enrolled in Phase I to III lapatinib clinical trials, 1.6% of patients experienced a significant decline in LV systolic dysfunction, with 0.2% being symptomatic [16]. For patients who were previously treated with anthracyclines, trastuzumab, or neither prior to lapatinib therapy, the incidence of cardiac events was 2.2%, 1.7%, and 1.5%, respectively [16]. The mean time to the onset of cardiac events was 13 weeks, and 88% of patients followed had a partial or full recovery of LV function regardless of continuation or discontinuation of lapatinib [16]. QT interval prolongation (QTc > 480 ms or an increase in QTc > 60 ms from baseline) was not found to be significant in a retrospective study, although the group analyzed only included 16 patients, and one patient increased in common terminology criteria for adverse events [17]. Nevertheless, other studies have found it to occur in up to 16% of patients [6]. Experiments using a whole-cell patch clamp suggest that this arises from the human Ether-à-go-go-related gene (hERG) channel current delay, which slows heart repolarization. This seems to arise from the inhibition of the coding of the main IK_r_ current pore-forming subunit [18].

Erlotinib and Gefitinib

EGFR is overexpressed and/or mutated in many solid tumors [3]. Erlotinib and gefitinib are orally active TKIs targeting EGFR and are used in the treatment of refractory, locally advanced, or metastatic non–small-cell lung cancer after platinum-based chemotherapy [19-20]. Non-smokers, females, Asian descent, and adenocarcinoma phenotype (bronchioalveolar type, in particular) are preferred candidates for these agents [21-23]. Erlotinib is also approved for the treatment of pancreatic cancer in combination with chemotherapy.

In a study of pancreatic cancer patients administered gemcitabine with or without erlotinib, myocardial ischemia or infarction occurred in 2.3% of patients receiving both and 1.2% of those who received gemcitabine alone [19]. An increased incidence of deep venous thrombosis (DVT) with erlotinib and gemcitabine combination therapy was also noted (3.9% vs 1.2% with gemcitabine, respectively). The mechanism by which these agents contribute to venous thromboembolic events (VTE) remains to be elucidated, but a meta-analysis suggests that the possible mechanism that associates anti-EGFR drugs and thrombosis events is linked to the anti-angiogenic effect of this inhibition. This blockage results in a decrease in production of angiogenic factors, such as VEGF, which enhances the production of nitric oxide (NO). This, in turn, has antiplatelet action and inhibition of leukocyte adhesion that has the potential to expose prothrombotic phospholipids and lead to thrombosis [24].

Additionally, it is important to consider that both erlotinib and gefitinib are agents that are used after platinum-based chemotherapy. A meta-analysis determined that of 932 patients in cisplatin chemotherapy analyzed, 18.1% developed a thromboembolic event. Of these, 49.7% exhibited deep vein thrombosis [25]. Thus, it cannot be discarded that there is a possibility that some of the thromboembolic risk attributed to the mentioned TKIs can arise from a predisposition generated by the previous treatment with platinum-based chemotherapy. It has been demonstrated in-vitro that cisplatin has the potential to increase platelet count and endothelial cell damage, which can increase thrombotic events, especially when coupled with gemcitabine treatment [26].

Sunitinib

Sunitinib is an orally active, multitargeted, antiangiogenic small molecule TKI that inhibits vascular endothelial growth factor receptor (VEGFR), c-Kit, PDGFR-α and -β, rearranged during transfection (RET) receptor, and FMS-like tyrosine kinase 3 (FLT-3) receptor [27]. It is considered the standard-of-care for treatment of renal cell carcinoma and as a second-line treatment for patients with GIST refractory to imatinib monotherapy [28].

Sunitinib-induced cardiotoxicity is well recognized and the cause of considerable cardiac morbidity (i.e., hypertension, heart failure, myocardial ischemia, etc.) that may not be manifest for weeks or months after completion of sunitinib therapy [29]; the mean time to development of heart failure is highly variable, ranging from 22 days to 27 weeks post-therapy [30-31].

Sunitinib inhibits a large number of “direct targets” and “off-targets”, which makes it difficult to determine the specific pathway(s) leading to cardiotoxicity. The cardiotoxic effects are mediated, at least in part, through inhibition of PDGFR-β; microscopic findings compatible with toxin-induced myopathy and mitochondrial damage are present in endomyocardial biopsies from patients with sunitinib-induced heart failure [30]. Cardiomyocyte PDGFR-β expression and activity increase in response to pressure overload and regulate myocardial angiogenesis, with PDGFR-β knock-out animal models exhibiting impaired stress-induced angiogenesis, myocardial contractile dysfunction, and heart failure [32]. “Off-target” inhibition of AMP-activated protein kinase, ribosomal S6 kinase, and a tyrosine kinase receptor by sunitinib leads to maladaptation to pressure overload (i.e., systemic hypertension), myocyte adenosine triphosphate (ATP) depletion and apoptosis [3, 31]. Maladaptation to pressure loading may be particularly important as hypertension is a common side effect of sunitinib and other VEGFR inhibitors [31]. The mechanism whereby VEGFR antagonists cause hypertension and heart failure is decreased capillary permeability leading to increased cardiac afterload (see also bevacizumab) [33].

LV systolic dysfunction and heart failure are reported in an interval from 3% to 18% [34] and symptomatic heart failure in 3% to 15% [30-31, 35] of sunitinib-treated patients, with the variability due to the heterogeneous patient populations. Most of these patients with sunitinib-induced cardiotoxicity have coronary artery disease as their only risk factor for heart disease. In clinical studies, heart failure symptoms occurred 22 to 435 days after initiation of sunitinib therapy (average 30.5 weeks) and usually responded well to discontinuation or dose modification of sunitinib and initiation of routine heart failure medical therapy. However, in some individuals, myocardial dysfunction was not reversible despite appropriate therapy.

Other cardiovascular complications associated with sunitinib therapy include elevated serum troponin levels (18%), MI (2.4%), and transient ischemic attack (1%) [30- 31]. Approximately one-fourth of sunitinib-treated patients develop systemic hypertension (> 150/100 mmHg) with severe (Grade 3 or higher) hypertension occurring in 2% to 12% [30, 33, 36]. If hypertension develops, it usually does so with the first cycle of sunitinib and persists throughout treatment [30].

Sorafenib

Sorafenib is a multitargeted, small molecule TKI that inhibits pathways important in cellular proliferation (i.e., RAF-1, B-type Raf [BRAF], and c-Kit) and pathways that are pivotal in tumor angiogenesis (i.e., VEGFR-2, VEGFR-3, FLT-3, and PDGFR-β) [37]. It is currently indicated as a second line for the treatment of renal cell and hepatocellular carcinoma, and as such, it is frequently administered after sunitinib therapy, which raised concerns of cardiotoxicity potentiation [38]. However, a retrospective analysis of 68 patients treated with sorafenib following sunitinib treatment did not reveal increased cardiotoxicity rates with sequential administration [39].

The incidence of acute coronary syndromes, including MI, in patients treated with sorafenib, has been reported to occur in 2% to 3% of such individuals [38-42]. In an observational study of 74 patients with metastatic renal cell carcinoma who received either sunitinib, sorafenib, or both by sequential administration, 34% experienced a cardiac event -- defined as the occurrence of increased cardiac enzymes, symptomatic arrhythmia requiring treatment, new LV systolic dysfunction, or acute coronary syndrome, 40% had electrocardiographic changes, and 18% were symptomatic, with 9% of patients seriously compromised and requiring intermediate care and/or intensive care admission [43].

The pathophysiology of sorafenib-associated cardiotoxicity may be explained by VEGFR and PDGFR inhibition [33, 44-46]. Additionally, RAF inhibition may play a role in its toxicity profile. RAF1 is a member of the RAF family of intracellular signal transducing kinases that inhibit proapoptotic kinases -- MST2 and apoptosis signal-regulating kinase 1 (ASK1) – that regulate oxidant stress-induced injury [47-49]. RAF1 gene deletion in cardiomyocytes results in the development of a dilated, hypocontractile heart in animal models [49].

A meta-analysis of 4,599 sorafenib-treated patients reported an overall incidence of hypertension of 23% with the frequency of Grade 3 or 4 hypertension ranging from 2% to 31% [50]. When studied prospectively, a persistent increase in blood pressure was observed within three weeks of sorafenib treatment in most patients, and vascular stiffness increased significantly for up to 10 months of observation [51]. VTE has been associated with sorafenib administration, but the incidence is low (< 2%) [33, 52].

Cabozantinib

Cabozantinib is a tyrosine kinase inhibitor that is Food and Drug Agency (FDA) approved for the treatment of renal cell carcinoma that has been previously treated with anti-angiogenic therapy. It has inhibitory action over VEGFR2 and tyrosine protein kinase Met (c-MET) [53].

A meta-analysis that revised the development of hypertension in prospective trials with cancer patients following cabozantinib treatment found an incidence of 27.8% (95% confidence interval (CI): 23.2 - 32.8%) for all grade hypertension and 12.0% (95% CI: 10.2 - 14.1%) for high-grade. It is suggested that this occurs through the interaction with VEGFR 2 and VEGF-induced vasodilatory and hypotensive effects. Contrariwise, inhibition of this pathway can produce a hypertensive response [53].

An FDA risk assessment report for cabozantinib indicated that, during the clinical testing program for this drug, no patients suffered torsades des pointes nor were the QTcF > 500 reported [54]. Another FDA risk assessment report wrote that arterial and venous thromboembolism occurred with the administration of cabozantinib in 2% and 6% incidence, respectively [55].

Dabrafenib and Trametinib

Dabrafenib and trametinib are serine-threonine kinase inhibitors that work by blocking the BRAF (dabrafenib and trametinib) and MEK (trametinib only) kinases, which then signal the ERK pathway [56]. Their combined use is FDA approved for the treatment of metastatic melanoma previously diagnosed by an FDA-approved test that detects a mutation in BRAF V600E or V600K. They have a synergistic effect arising from the targeting of different stages of the pathway [57].

Nevertheless, these inhibitors show a series of cardiotoxic complications with their use. For instance, a review that reports on the safety of TKIs establishes that, across clinical trials, evidence for cardiomyopathy (left ventricle dysfunction as measured by the decrease of LVEF > 10% below baseline) was 11% when trametinib was administered as a single agent (n = 329) and 8% when combined with dabrafenib (n = 202). It also mentions that a study found that the incidence was 9% (5/55) with the combination therapy, while dabrafenib alone showed an incidence of 0%. This suggests that the adverse cardiotoxic effects of dabrafenib might appear only in combination therapy. Correspondingly, this exhibits an average onset of 63 days (range: 16 - 156 days) for single trametinib administration and 86 days (range: 27 - 253 days) for combined treatment [58].

Comparably, the incidence of hypertension (all grades) was found to be 4% in a clinical trial performed on the adverse effects of combined trametinib and dabrafenib therapy. Crossing over from a trametinib only treatment to a combined one in the same trial led to an incidence of 9% for all grades of hypertension and 7% for Grades 3 or 4 [59].

It is suggested that the cardiotoxic effects of these drugs and other BRAF inhibitors arise from blocking of the activation via growth factor of the ERK pathway in cardiomyocytes. This was investigated in perfused rat hearts [56].

Lenvatinib

Lenvatinib is recognized as a multi-targeted tyrosine kinase inhibitor. Its primary activity is over vascular endothelial growth factor receptor (VEGFR), which has an anti-angiogenic effect. This effect accounts for it being FDA approved as a second-in-line treatment for metastatic and progressive thyroid cancer that is refractory to radioiodine (iodine-131), after receiving previous anti-angiogenic therapy. Moreover, it also inhibits other molecular pathways of tumor growth, such as platelet-derived growth factor receptor (PDGFR) and the fibroblast growth factor receptor (FGFR) [60].

One of the major cardiac incidents reported in the use of lenvatinib is the high incidence of hypertension in clinical trials. For instance, a study of this type recognized the appearance of this condition in 69.3% (n = 261) of the subjects for all grade hypertension and 42.9% for Grades 3 and above [60]. This consequence might arise from a similar mechanism than in cabozantinib administration, where the VEGFR is also inhibited. Less common, although severe, effects reported for the use of lenvatinib in clinical trials were cardiac dysfunction, with a reported incidence of 2% against 0% in placebo, and arterial thrombosis, reporting an incidence of 5% against 2% placebo (lenvatinib n = 261; placebo n = 131) [61].

Ponatinib

Ponatinib is indicated in chronic myelogenous leukemia (CML), which is intolerant or resistant to previous treatments. Ponatinib is also used for the treatment of Philadelphia-positive acute lymphoblastic leukemia (ALL) that has the T315I mutation and are resistant to prior therapy with TKIs, such as dasatinib or nilotinib [62-63]. This drug is a third generation inhibitor of the BCR-ABL receptor; its potency relies on the fact that it has clinical activity on both the wild-type and mutated (for example, in CML) BCR-ABL, including the T315I mutation [64].

The two predominant clinical manifestations reported in trials for the use of ponatinib were hypertension and arterial thrombotic events (ATEs). In the case of the first condition, a clinical trial - which involved the analysis of patients with chronic-phase (n = 270), accelerated-phase (n = 85), and blast-phase (n = 62) CML, and Ph-Positive ALL (n = 32) –  determined an incidence of 9%, 7%, 2%, and 3% of all-grade hypertension for each type of cancer, respectively, and 2%, 4%, 2%, and 3%, also respectively, for Grade 3 or 4 hypertension [65]. In a similar manner, a trial that evaluated the efficiency and safety of ponatinib in 449 CML or ALL (Ph-positive) patients describes that ATEs were present in 19% of patients, including 10% cardiovascular, 7% cerebrovascular, and 7% peripheral vascular events. Additionally, it reports that venous thromboembolic events were observed in 5% of the patients. It might be important to mention that this trial had to be temporarily interrupted due to the appearance of ATEs, requiring ponatinib dosage modification before continuing [66].

Furthermore, ponatinib is a TKI that had to be provisionally removed from the market until modifications in the labeling for the safety of thromboembolic events and arterial occlusion were included before its reintroduction [58].

### Epigenetic modulators

**Figure 2:**
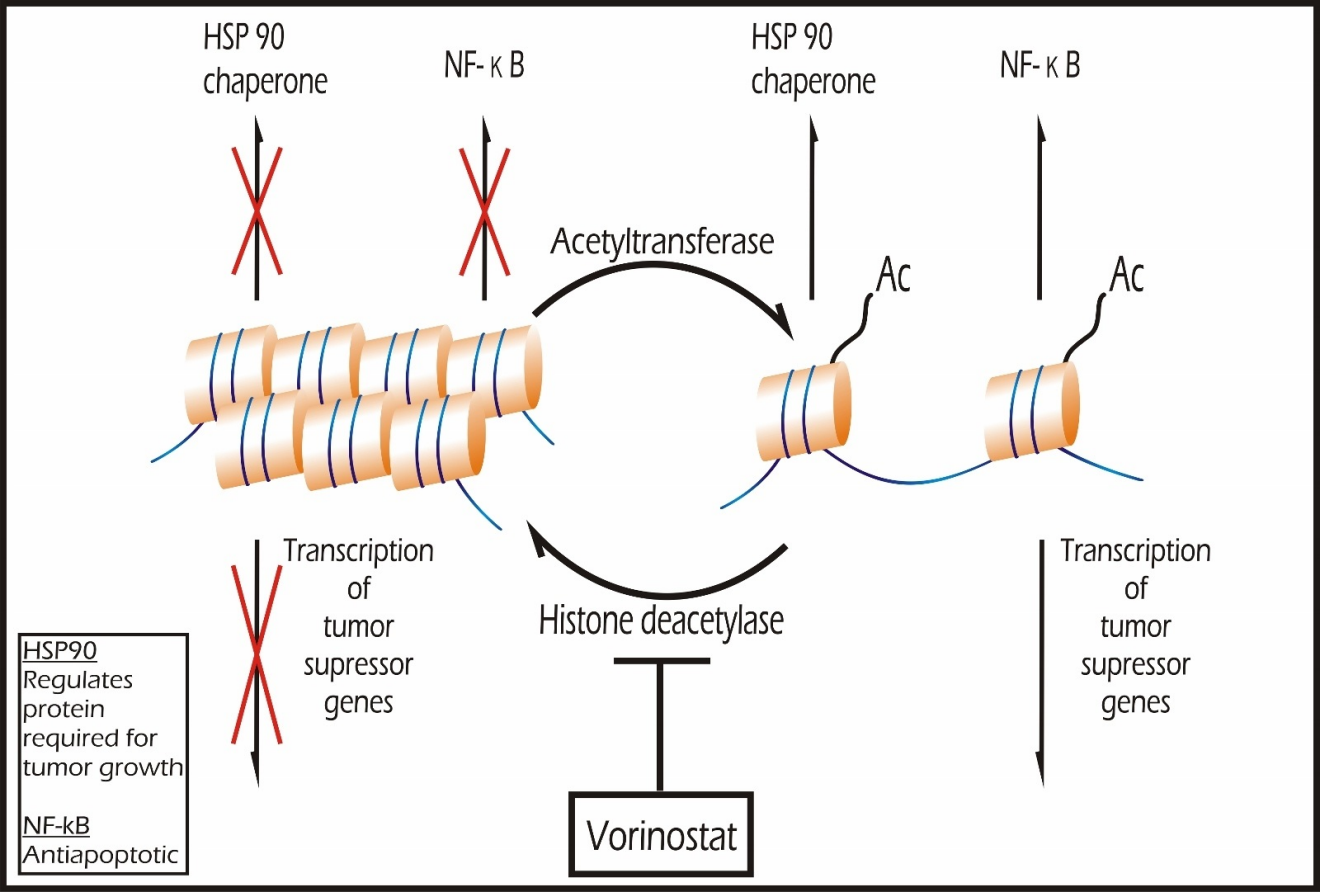
Epigenetic Modulators Histone deacetylase (HDAC) and acetyltransferases are "epigenetic agents" that regulate the acetylation of histone proteins, thereby activating chromatin transcription at specific gene loci. They also regulate the acetylation of non-histone proteins, including transcription factors involved in cell cycle progression and apoptosis. HDAC inhibitors can favorably affect transcription patterns in cancers that exhibit aberrant acetylation patterns that result in (a) transcriptional silencing of tumor suppressor genes; (b) inactivation of heat shock protein (HSP) 90 chaperone function; and/or (c) abnormal nuclear factor kappa B (NF-kB) signaling.

Histone deacetylase (HDAC) and acetyltransferases are “epigenetic agents” that regulate the acetylation of histone proteins, thereby activating chromatin transcription at specific gene loci. They also regulate the acetylation of non-histone proteins, including transcription factors involved in cell cycle progression and apoptosis [67-69]. HDAC inhibitors can favorably affect transcription patterns in cancers that exhibit aberrant acetylation patterns that result in (a) transcriptional silencing of tumor suppressor genes [70-71]; (b) inactivation of heat shock protein (HSP) 90 chaperone function; and/or (c) abnormal nuclear factor kappa B (NF-κB) signaling [72] as shown in (Figure 2).

Vorinostat

Vorinostat (suberoylanilide hydroxamic acid, SAHA) is an HDAC inhibitor that is currently FDA approved for the treatment of recurrent cutaneous T-cell lymphoma (CTCL) and is under investigation for other (i.e., hematologic) malignancies. In the cardiomyocyte, HDAC influences cardiac hypertrophy, and epigenetic modifications may contribute to cardiac dysfunction and heart failure [73-75]. In dilated cardiomyopathy-derived myocardial human tissue, epigenetic changes in several signaling regulatory pathways have been demonstrated [76].

In patients without known heart disease, vorinostat therapy has been associated with dyspnea in 32% to 47% of patients, QT interval prolongation in 3.5% to 6%, and thromboembolic events (DVT or pulmonary embolism) in 5% to 8% [77-81]. Results of ongoing studies of vorinostat and other cancer therapies targeting epigenetic modifiers are needed to provide further information regarding their cardiotoxicity profile.

### Proteasome inhibitors

Bortezomib

**Figure 3:**
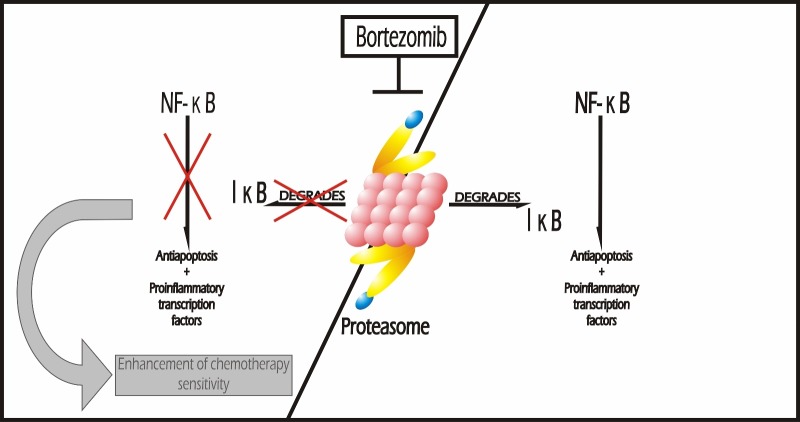
Proteasome Inhibitors The main target of bortezomib is the ubiquitin-proteasome system (UPS). Bortezomib inhibits proteasomal degradation of IkB-alpha, leading to suppression of the antiapoptotic and proinflammatory transcription factor NF-kN and subsequent enhancement of chemotherapy sensitivity.

Bortezomib (PS-341) is the first proteasome inhibitor approved by the FDA for the treatment of malignancies. It exhibits antiproliferative and proapoptotic effects on plasma cells and is approved for initial treatment of patients with multiple myeloma [82-83]. As shown in Figure 3, the main target of bortezomib is the ubiquitin–proteasome system (UPS), which is a lysosome-independent cellular protein degradation system involved in the regulation of protein expression signaling and proliferation of malignant cell lines [84-86]. Bortezomib also inhibits proteasomal degradation of IκB-alpha, leading to the suppression of the antiapoptotic and proinflammatory transcription factor, NF-κB, and subsequent enhancement of chemotherapy sensitivity [73]. Figure 3 represents the mechanism of action of this drug and its role in the enhancement of chemotherapy sensitivity.

Although case reports of bortezomib-induced cardiotoxicity have been published [87-89], the frequency has not been determined. In a Phase III clinical trial of 669 multiple myeloma patients comparing bortezomib with high-dose dexamethasone, the incidence of cardiovascular complications was 15% versus 13%, respectively, with 2% of patients in both treatment groups experiencing heart failure [90]. Other proteasome inhibitors (i.e., NPI-0052, CEP-18.770, and RP-171) are currently in early phase trials may help to elucidate the clinical impact of UPS disruption on the cardiovascular system [73, 91].

Patients suffering from subclinical heart disease are thought to be at particularly increased risk for developing cardiotoxicity with bortezomib treatment [73, 88]. In animal models, UPS is critical for the maintenance of normal cardiac physiology [85, 88], with its impairment leading to the accumulation of oxidized ubiquitinated proteins, which promote cardiomyocyte death and myocardial dysfunction [92]. In ischemia/reperfusion injury models, UPS may be activated as an adaptive mechanism to preserve myocyte and cardiac function [93].

### Monoclonal antibodies

Bevacizumab

Bevacizumab is a chimeric, monoclonal antibody [94] that binds to biologically active isoforms of vascular endothelial growth factor A (VEGF-A), thereby preventing interaction with its endothelial cell receptors (Fms-related tyrosine kinase 1 (FLT-1) and kinase insert domain receptor (KDR)). Bevacizumab is FDA approved for the treatment of various malignancies: it is used in combination with intravenous 5-fluorouracil-based (5-FU) chemotherapy for(a) for first- or second-line treatment of patients with metastatic carcinoma of the colon or rectum; (b) in combination with carboplatin and paclitaxel for the first-line treatment of patients with unresectable, locally advanced, recurrent or metastatic non-squamous non-small cell lung cancer; and (c) for the treatment of metastatic renal cell carcinoma in combination with interferon alfa. It is also approved, in combination with paclitaxel, for the initial treatment of patients with metastatic human epidermal growth factor receptor 2 (HER2) negative breast cancer. Lastly, it has recently been approved for treatment of glioblastoma, as a single agent for patients with progressive disease following prior therapy [95].

As with other anti-VEGF targeted therapies, hypertension is a common adverse effect in patients treated with bevacizumab monotherapy or in combination with other targeted agents. In clinical trials, the incidence of bevacizumab-induced hypertension has been reported between 4% to 35% [6, 33, 96-103], with Grade 3 hypertension reported in 11% to 18% of patients [6, 96-98, 100, 104-105] and approximately 2% of patients having severe (Grade 4) hypertension requiring discontinuation of the drug [106]. The median interval from the initiation of bevacizumab therapy to the development of hypertension (HTN) is 4.5 to 6 months [33]. Interestingly, the development of hypertension during bevacizumab therapy is considered a favorable prognosticator, as it denotes the presence of certain “hypertension-susceptible” VEGF polymorphisms (VEGF-2578 AA and VEGF-1154 A) linked to a better response to chemotherapy and increased survival [107]. Higher doses and concomitant therapy with sorafenib are associated with an increased incidence of hypertension [33, 108-109].

Cardiac dysfunction and heart failure are potential adverse effects of therapy with bevacizumab, with a reported incidence of heart failure ranging from 1.7% to 3% [95-97]. Patients previously exposed to traditional therapies known to cause cardiomyopathy, such as anthracyclines [96, 110], mitoxantrone [111], or capecitabine [96], are at particular risk of suffering this complication through potentiation of their cardiotoxic effects.

The mechanism of bevacizumab therapy-related HTN is thought to be related to microvascular rarefaction and inhibition of the nitric oxide-mediated vasomotor effects of VEGF. Microvascular rarefaction is an anti-angiogenic effect leading to the extinction of the small arterioles and capillaries that comprise the microcirculation, and it is a common finding present in individuals suffering from arterial hypertension [112]. The presence of this phenomenon associated with bevacizumab therapy was documented in a prospective study demonstrating a decline in the mean dermal capillary density after six months of treatment and its association with the development of hypertension [113].

Inhibition of VEGFR-2 activation with bevacizumab leads to blockage of endothelial nitric oxide synthase up-regulation through the Src and Akt signaling pathways. The resultant decreased nitric oxide production promotes vasoconstriction and increases peripheral vascular resistance [114].

Bevacizumab-induced heart failure is closely related to uncontrolled hypertension and the cardiac remodeling response. VEGF signaling in cardiomyocytes is a major mediator, not only in angiogenesis but also in compensatory responses to pressure load and injury [33, 115-116]. In animal models mimicking bevacizumab anti-VGEF effects, pressure overload results in the reduction of myocardial capillary density, global contractile dysfunction, cardiac fibrosis, and eventually decompensated heart failure [117].

Venous and arterial thromboembolic events, including angina pectoris, myocardial ischemia, or infarction and cerebral infarction, occur at a higher incidence in patients treated with bevacizumab, plus chemotherapy, as compared with those treated with chemotherapy alone [95]. A meta-analysis of 15 randomized trials demonstrated a 33% increased risk of developing VTE associated with bevacizumab treatment (relative risk: 1.33; p < .001) [118]. A pooled analysis of five randomized clinical trials demonstrated an incidence of arterial thrombotic events of 3.8% in the bevacizumab-treated patients and 1.5% developed myocardial infarction or ischemia [119]. An observational study reported serious arterial thrombotic events in 1.8% and MI in 0.6% of bevacizumab-treated patients [120]. These events tend to occur at any time during therapy, with a median time-to-event of about three months and are not dose-dependent. A history of prior vascular thrombosis and age > 65 years have been identified as potential risk factors [119-120].

VEGF plays a significant role in the maintenance of vascular integrity through the stimulation of endothelial cell proliferation and preservation of endothelial cell junctions [121]. VEGF inhibition with bevacizumab promotes endothelial cell dysfunction and apoptosis and decreases the endothelial regenerative potential, which predisposes to both hemorrhagic and thrombotic events, especially in the setting of trauma [114, 121]. Platelet activation and aggregation due to subendothelial collagen exposure and subsequent tissue factor activation are key factors in the prothrombotic cascade [114]. Additionally, reduction of nitric oxide and prostacyclin promote vasoconstriction and thrombosis [33, 114].

Trastuzumab

Trastuzumab is a chimeric, monoclonal IgG antibody against the extracellular domain of HER2 [122-123]. HER2 protein overexpression is observed in 20% to 35% of primary breast cancers [122-125] and is associated with poorer outcomes [124]. Trastuzumab, as a single agent or in combination with immunochemotherapy, improves outcome in breast and gastroesophageal cancer patients who overexpress HER2 [124, 126]. The risk of recurrence and mortality are reduced when trastuzumab is integrated into adjuvant chemotherapy for early stage localized breast cancer that overexpresses HER2 [124, 127]. As a result, many breast cancer patients who are treated with trastuzumab receive anthracyclines before or simultaneously. 

LV systolic dysfunction is the most common cardiotoxic effect induced by trastuzumab, with the mechanism, pathologic findings, and clinical outcome different than anthracycline-induced cardiac dysfunction. The cardiotoxic effects of the trastuzumab are not cumulative or dose-related, as seen with anthracyclines. Although the risk of cardiomyopathy is increased in patients who have been treated with both agents, some develop heart failure during treatment with trastuzumab in the absence of exposure to anthracyclines [125, 127-129]. Endomyocardial biopsies have revealed two types of chemotherapy-induced cardiac dysfunction (Table 1) [128-129]. Type I cardiotoxicity is characteristic of anthracycline exposure with myocyte damage on pathologic biopsy, clinical heart failure, and minimal or no improvement in ventricular function with cessation of therapy. Type 2 cardiotoxicity is characterized by reduced contractility with little myocyte necrosis on microscopic examination and frequent improvement in ventricular function with cessation of therapy [128].

**Table 1:** Clinical Features Distinguishing Type I and Type II Chemotherapy- related Cardiac Dysfunction (CRCD) [128, 130]

Type I CRCD (model: doxorubicin)	Type II CRCD (model: trastuzumab)
Cellular death	Cellular dysfunction
Myocyte necrosis and typical ultrastructural changes on light and electron microscopy	No injury or myonecrosis by light and electron microscopy
Cumulative dose-related	Not cumulative dose-related
Permanent damage	Generally reversible with cessation of drug

Endomyocardial biopsies in patients with trastuzumab-induced LV dysfunction show no light or electron microscopic evidence of injury [5, 128], suggesting that trastuzumab depletes adenosine triphosphate by impairing mitochondrial function without permanently altering myofibrillar ultrastructure [131]. Alternatively, the cardiotoxicity associated with HER2 receptor blockade may result from a decreased ability to mount an integral response to stress [127-128]. Signal transduction via epidermal growth factors is fundamental in regulating the hypertrophic response to myocytes and the sarcomeric organizational response towards different stimuli, including protection against cardiac toxins [127-128, 132]. The HER2 gene knock-out mice have a higher sensitivity for anthracycline-associated cardiotoxicity and the development of progressive heart failure and premature death compared to wild-type mice [127-128, 133].

It is postulated that trastuzumab induces cardiotoxicity in hearts susceptible to dysfunction as a result of prior or concomitant anthracycline treatment (the so-called “two-hit theory”) [132] by interfering with the repair of myocytes damaged by anthracycline exposure.

In a pooled analysis of 1,219 patients enrolled in Phase II and III clinical trials, LV systolic dysfunction was noted in 9.2% of those who received trastuzumab, and the incidence was increased when trastuzumab was administered concurrently with anthracyclines. Severe heart failure symptoms (New York Heart Association (NYHA) Class III to IV) were present in 16% of trastuzumab-treated patients who received an anthracycline concomitantly and only 2% of those who received paclitaxel concomitantly [127, 134]. Of the patients who developed symptomatic heart failure with trastuzumab therapy, LV function normalized in 79% when the agent was discontinued and appropriate heart failure therapy initiated. Cardiotoxicity reversed quickly (average: 1.5 months) [134]; when trastuzumab therapy was reinitiated in 25 patients, only three (12%) had a recurrence of LV dysfunction [5, 135].

A pooled analysis of randomized controlled trials and case-control studies showed that the prevalence of cardiotoxicity in the trastuzumab-treated patients was 10% whereas, in the non-trastuzumab comparator arm, the prevalence was 2% [136]. In a meta-analysis of five randomized controlled trials, a 10% decline in LV ejection fraction was observed in 3% to 34% of trastuzumab-treated patients [128, 137].

Independent risk factors for trastuzumab-induced cardiotoxicity are simultaneous or prior exposure to anthracycline and increased patient age [6, 125, 128, 138]. Similar to anthracycline-induced cardiotoxicity, previous cardiac disease and NYHA Class II symptoms are suspected risk factors [6]. However, traditional cardiac risk factors, prior cardiac disease, prior chest radiation, and preexisting hypertension have not been identified as risk factors for trastuzumab-induced cardiac dysfunction [6, 128]. In patients receiving concurrent anthracycline and trastuzumab therapy, the risk of cardiac dysfunction increases after the cumulative dose of doxorubicin exceeds 300 mg/m^2^ [6, 127, 137-138].

Rituximab

Rituximab is a chimeric murine/human monoclonal antibody that binds the cluster of differentiation 20 (CD20) protein, which is expressed on the surface of B cells [139]. CD20 functions as an ion channel essential for regulating cell cycle progression and calcium homeostasis. Stimulation of the CD20 receptor induces depletion of intracellular calcium stores, thereby affecting calcium-dependent signaling processes, such as transcriptional control, cell cycle progression, and apoptosis [139-140].

Rituximab is indicated for the treatment of various non-Hodgkin’s lymphomas, either alone or in combination with other chemotherapeutic agents [141-142]. The major cardiovascular side effect observed with Rituximab therapy is hypotension, which occurs in up to 10% of patients [125]. It typically occurs in the first few hours of the drug’s initial infusion and is responsive to fluid therapy [125, 141-143]. The exact mechanism in which the cardiovascular system is affected is unknown, but it is likely related to rituximab’s calcium channel blocking function. Despite the acute effects, there is no increased risk of cardiotoxicity in patients with non-Hodgkin’s lymphoma when rituximab is added to standard (i.e., CHOP - cyclophosphamide, hydroxydaunorubicin, oncovin, and prednisone) chemotherapy [143].

Alemtuzumab

Alemtuzumab, a humanized IgG1 directed against the CD52 protein, is primarily indicated in patients with chronic lymphocytic leukemia (CLL) or small cell lymphoma [125, 144-145]. It is also used in patients with immune-mediated, nonmalignant conditions, such as rheumatoid arthritis, solid organ transplants, multiple sclerosis, and as a conditioning agent for bone marrow transplantation [144].

Alemtuzumab has been associated with infusion-related reactions, including hypotension, bronchospasm, and rash, typically during the first week of therapy [125, 142]. LV dysfunction is rare but has been reported in patients with cutaneous T-cell lymphoma who had previously received multiple chemotherapy regimens [125, 146]. The mechanism is not fully understood [146]. Close monitoring for hypotension is recommended for patients with the preexisting cardiac disease who are treated with this agent [125].

Ibritumomab Tiuxetan

Ibritumomab tiuxetan is an agent used in patients with relapsing or refractory low-grade follicular transformed B-cell non-Hodgkin’s lymphoma [144]. It is composed of an anti-CD20 mouse antibody (i.e., ibritumomab) chemically attached to a chelator linked to the beta-emitting isotope yttrium90 [147-148].

Hypotension and cardiac arrhythmias are rare complications associated with ibritumomab infusion [125, 142]. Since ibritumomab tiuxetan is administrated in combination with rituximab [147], it is unknown if the adverse cardiovascular reactions are the result of one or the other agents or their interaction. Additionally, the long-term cardiac effects of local beta-irradiation are unknown.

Tositumomab

Tositumomab is an IgG2a anti-CD20 monoclonal antibody derived from immortalized mouse cells. It is administrated in a sequential infusion followed by iodine131 (131I) tositumomab (i.e., the antibody linked to I131 by a covalent reaction) which emits both beta and gamma radiation [149-150]. It is indicated for the treatment of patients with CD20 antigen-expressed refractory, low-grade, follicular or transformed non-Hodgkin’s lymphomas, and in patients with rituximab-refractory non-Hodgkin’s lymphomas [144, 149].

Hypotension (7%), peripheral edema (9%), chest pain (7%), and vasodilatation (5%) are cardiovascular complications that have been described with the use of this antineoplastic compound [149]. Due to its radioactive emissions, studies assessing the potential cardiovascular effects of this radio-immunotherapeutic agent are still needed.

Cetuximab

Cetuximab, a human/mouse chimeric monoclonal IgG1 antibody that binds to human EGFR, is currently used to treat colorectal [125, 144, 151] and head and neck cancer [151]. Cetuximab blocks phosphorylation and activation of receptor-associated kinases, resulting in the inhibition of growth and survival of tumor cells that overexpress EFGR [152].

Potentially fatal infusion reactions involving severe hypotension have been described in approximately 3% to 5% of patients receiving this medication [125, 142, 144].

Panitumumab

Panitumumab is a recombinant human IgG2 kappa monoclonal antibody to EGFR [153] that has been approved for EGFR-expressing metastatic colorectal carcinoma with disease progression on or following fluoropyrimidine, oxaliplatin, and irinotecan-containing chemotherapy regimens.

Panitumumab and cetuximab have the same target receptor but different IgG isotypes, which may convey different ligand affinities and cardiotoxicity profiles. Peripheral edema is the most common cardiovascular side effect, occurring in 12% of panitumumab-treated patients [153]. As the use of this agent increases in patients with RAS and BRAF wild-type colorectal cancers, the cardiotoxic effects common to other agents that target the EGFR ligand (e.g., erlotinib, lapatinib, etc.) could also be noted in these patients.

Ofatumumab

Ofatumumab is an IgG1-kappa monoclonal antibody that binds to the CD20 molecule resulting in B-cell lysis [154]. This agent is FDA approved for the treatment of patients with CLL refractory to fludarabine and alemtuzumab [154]. Its role in the treatment of follicular non-Hodgkin’s lymphoma, diffuse B-cell lymphoma, rheumatoid arthritis, and multiple sclerosis is currently under investigation.

Reported adverse cardiovascular reactions include peripheral edema (9%), hypertension (5%), hypotension (5%), and tachycardia (5%) [154]. The pathophysiology of these side effects may be related to its interaction with CD20-like ligands in noncancerous tissues.

Lenalidomide

Lenalidomide is a thalidomide analog possessing immunomodulatory and antiangiogenic properties [149]. Lenalidomide is FDA approved for the treatment of myelodysplastic syndrome associated with chromosome 5q deletion and multiple myeloma, in combination with dexamethasone, in patients who have received at least one prior therapy [155-156]. Its exact mechanism of action is not fully understood, but it inhibits cell proliferation and affects inflammatory cytokines in vitro [156].

The most common cardiovascular adverse reactions associated with this agent are peripheral edema (20% incidence), atrial fibrillation (2.9%), and VTE. The latter varies in incidence from 3% to 75% [6, 156]. A black box FDA warning is included in the package insert for this medication indicating that patients with multiple myeloma who receiving lenalidomide combination therapy may benefit from simultaneous thromboembolism prophylaxis or aspirin [157]. Administered as a single agent, it is not associated with an increased risk of thrombotic events [6, 158].

### Checkpoint inhibitors

Nivolumab and Pembrolizumab

**Figure 4:**
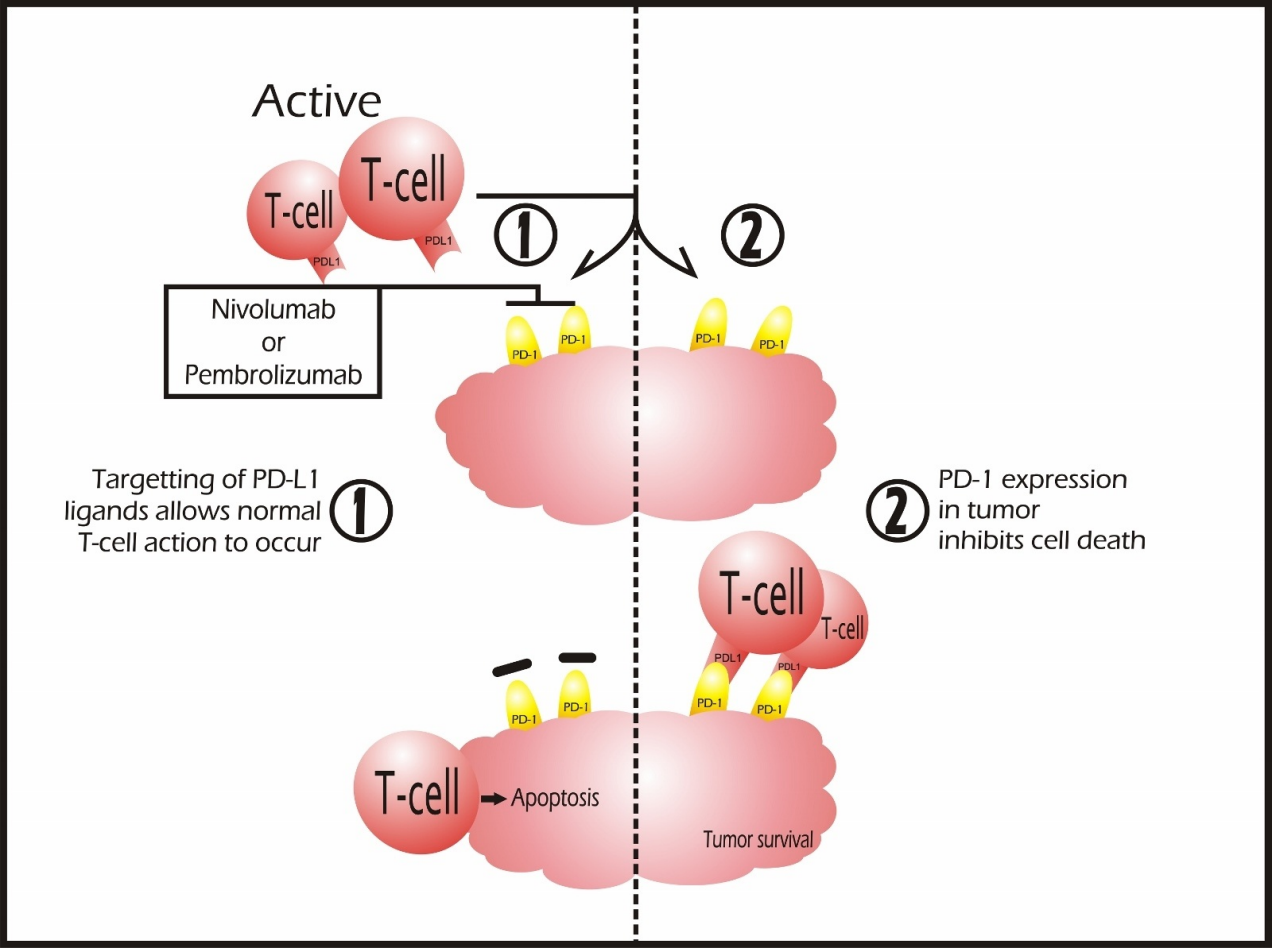
Checkpoint Inhibitors Nivolumab and pembrolizumab are two checkpoint inhibitors that work on PD-1 receptors to trigger T-cell activation. When T-cells reach cancer cells, inactivation of this pathway can happen due to the binding of the ligand PD-L1 to the mentioned receptor. Therefore, the use of these drugs serves as immunotherapy against tumor cells.

Nivolumab and pembrolizumab are two checkpoint inhibitors that work on PD-1 receptors to trigger T-cell activation. When T-cells reach cancer cells, inactivation of this pathway can happen due to the binding of the ligand PD-L1 to the mentioned receptor [159] as schematized in Figure 4. Therefore, the use of these drugs serves as immunotherapy against tumor cells. These are FDA approved for the treatment of (a) non-small cell lung cancer that has metastasized and (b) melanoma that cannot be removed by surgery. Additionally, nivolumab is approved for the treatment of classical Hodgkin lymphoma and advanced renal cell carcinoma.

The incidence of cardiotoxicity of checkpoint inhibitors is reported to be very low in early clinical trials. For instance, a review reported that only 2% of 296 patients who received 10 mg/kg of nivolumab exhibited hypotension. Similarly, only 7% of 135 melanoma patients who received 10 mg/kg of pembrolizumab showed a development of hypertension [160]. Cardiotoxic mechanisms are still to be elucidated, but mouse models suggest that heart dysfunction and dilation occur as an autoimmune response to cardiac troponin I in PD-1 deficient mice through chronic stimulation of Ca2+ influx [161]. It might be relevant to assess potential cardiotoxic effects from PD-1 ligand inhibition as treatment with these checkpoint inhibitors is expected to expand to several other cancer types.

### mTOR inhibitors

**Figure 5:**
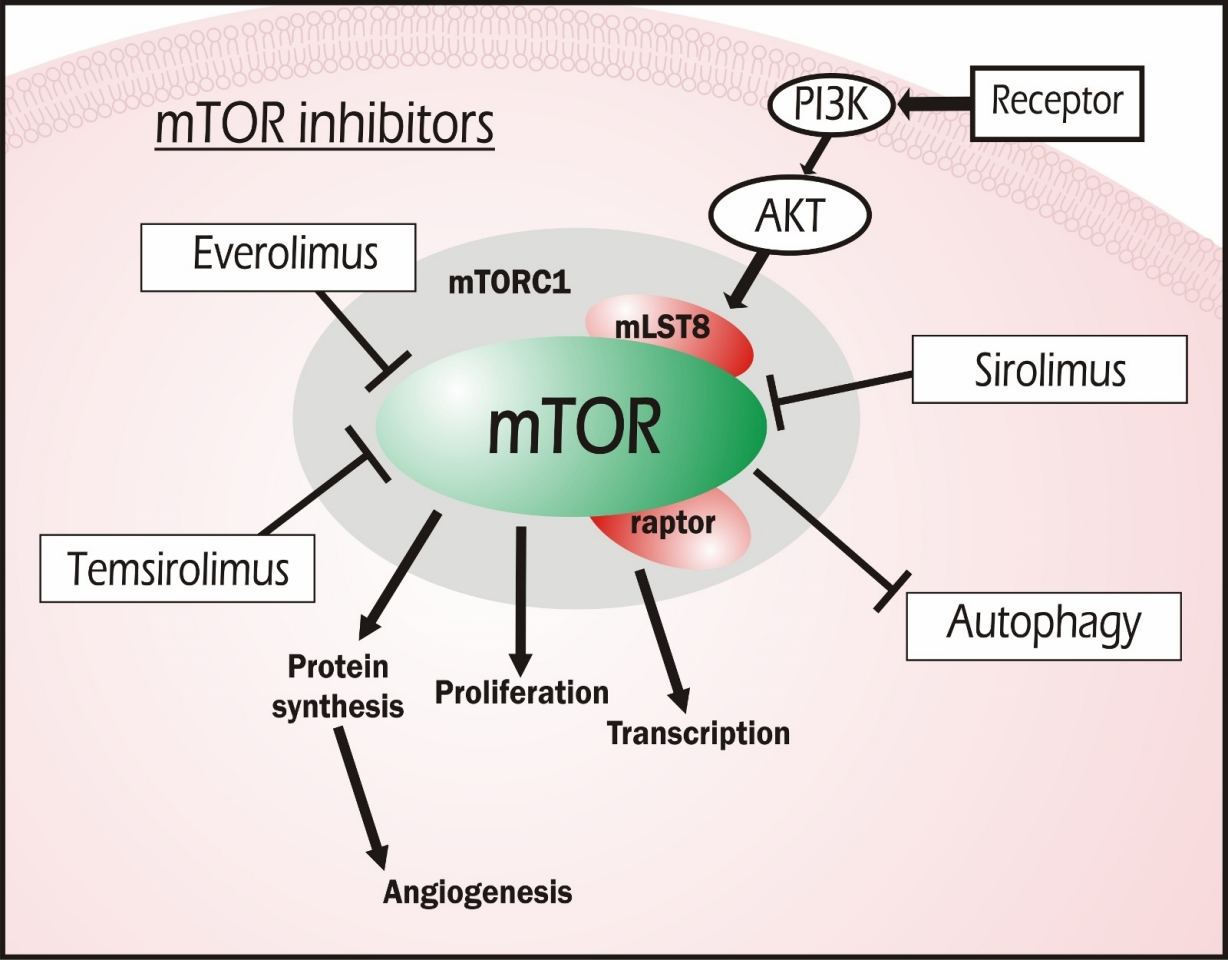
mTOR Inhibitors The target of these agents is the mammalian target of rapamycin (mTOR), a key regulatory kinase that interferes with cell cycle progression from the G1 to S phase via the formation of an immunosuppressive complex with FK binding protein 12 (FKBP-12). Inhibition of this pathway by everolimus, sirolimus, or temsirolimus reduces possible angiogenesis and proliferation of cells.

Figure 5 exhibits simplified pathways for the mammalian target of rapamycin and the roles it serves in cell cycle progression, transcription, and other factors related to proliferation. 

Sirolimus

Sirolimus inhibits (a) T-lymphocyte activation and proliferation in response to antigenic and cytokine stimulation and (b) antibody production by inhibiting the mammalian target of rapamycin (mTOR), a key regulatory kinase that interferes with cell cycle progression from the G1 to S phase, via the formation of an immunosuppressive complex with FK binding protein-12 (FKBP-12) [162-163]. This agent is used to prevent rejection in solid organ transplantation and treat Kaposi sarcoma in renal transplant patients.

Since this medication and its derivatives (discussed below) have a complex mechanism of action -- interacting with various growth factors, the redox state of the cell, transcription and protein synthesis, and cell survival -- the cardiovascular effects are also complex and not fully elucidated. In the mice model, mTOR expression preserves cardiac function by controlling collagen generation, attenuating fibrosis [164], and suppressing cytokine-mediated pathways responsible for cardiac dysfunction [165]. In animal models, rapamycin (or everolimus) interferes with post-MI LV remodeling through augmentation of autophagy [166].

Clinical studies have demonstrated dose-related cardiovascular effects in ≥ 20% of patients treated with rapamycin, including peripheral edema (in 54% to 64%), hypertension (in 39% to 49%), peripheral edema (in 16% to 24%), and chest pain (in 16% to 24%) of affected individuals. Severe cardiovascular effects -- atrial fibrillation, heart failure, DVT, and hypotension -- are uncommon, with a reported incidence of < 3% [162].

Everolimus

Everolimus, a derivative of sirolimus, is FDA approved for the treatment of patients with advanced renal cell carcinoma (RCC) after failed treatment with sunitinib or sorafenib and as prophylaxis for renal transplant rejection [167]. Through the formation of a complex with the FKBP-12 protein, it inhibits mTOR and subsequent phosphorylation of P70 S6 ribosomal protein kinase, thereby preventing protein synthesis and cell proliferation [167]. Additional antiproliferative effects are exerted by decreasing eukaryotic elongation factor 4E-binding protein and expression of VEGF and hypoxia-inducible factor [167].

In clinical trials, patients treated with everolimus experienced hypertension (4% of advanced renal cancer patients and 30% of kidney transplant recipients), peripheral edema (25% of advanced renal cancer patients and 45% of kidney transplant recipients), and tachycardia rarely [167].

Temsirolimus

Temsirolimus, also a derivative of sirolimus, leads to G1 phase cycle cell arrest and exerts antiangiogenic properties by reducing the synthesis of VEGF. It is FDA approved for the treatment of advanced renal cell carcinoma [168]. Cardiovascular side effects associated with its use include hypertension (7%), and VTE (2%) [168]. Figure 5 presents the mechanism of action of the mTOR inhibitors herein described.

### Prevention and treatment

Increased awareness of the potential cardiovascular side effects associated with the various chemotherapeutic agents allows early detection and appropriate treatment. Cardiac events associated with newer agents have a highly variable incidence and onset, ranging from days to months after the treatment is administered. Oncologists, cardiologists, and primary care physicians should educate patients about potential cardiotoxicity associated with chemotherapy, risk factors, the need for ongoing monitoring during administration of chemotherapy, and long-term follow-up to assess for late cardiovascular complications. Although the Heart Failure Society guidelines do not recommend reevaluation of cardiac function on a regular basis, monitoring of LV function at regular intervals should be strongly considered since many patients who develop decreased LV ejection fraction are asymptomatic [169]. Regular monitoring of cardiac function is particularly important in individuals considered to be at increased risk of chemotherapy-induced cardiotoxicity or those receiving an agent or agents with a high incidence of cardiotoxicity.

For patients receiving trastuzumab, some oncology centers propose that patients with elevated risk (i.e., elderly, reduced LV ejection fraction, cardiac risk factors present, etc.) undergo a clinical and echocardiographic assessment every three months while receiving chemotherapy and then every six months for five years subsequently [169-170]. For patients without elevated risk, the assessments can be performed every six months until the conclusion of trastuzumab therapy and yearly thereafter for three years. If new symptoms occur or if the LV ejection fraction declines by more than 10%, cessation of treatment – at least transiently -- may be necessary. If late cardiac complications related to trastuzumab are found, longer term monitoring may be appropriate [128, 171].

Newer noninvasive imaging modalities (i.e., speckle tracking echocardiography) may allow earlier detection of chemotherapy-induced cardiac dysfunction, even before frank systolic dysfunction occurs [172]. Efforts to identify biomarkers, such as serum cardiac isoenzymes, including troponin and/or brain natriuretic peptide, for early diagnosis of chemotherapy-induced cardiotoxicity and follow-up of this entity are also under investigation [128, 169, 171, 173].

Once a cardiovascular complication has been detected, efforts should be aimed to minimize the progression of cardiac and endothelial dysfunction. This may require pharmacokinetic changes, switching to chemotherapeutic analogs that are less cardiotoxic, administering cardioprotective agents (e.g., dexrazoxane), and avoiding additional cardiotoxic regimens. In many patients, discontinuation of the chemotherapeutic agent is the only available option, which might not be feasible or acceptable to the patient due to the risk of cancer progression. 

With regard to pharmacologic therapy, evidence-based management guidelines have not been established, although most experts initiate standard therapies based on presumed benefit. These treatments may include antihypertensive medications, diuretics, renin-angiotensin-aldosterone system (RAAS) blockers, beta-blockers, antiplatelet agents, or antiarrhythmics.

Treatment of hypertension should be aimed to reduce morbidity and mortality and lower the risk of associated end-organ damage. Hypertension induced by VEGF targeting agents is highly responsive to antihypertensive therapy, which means that interruption of chemotherapy is not usually necessary. Angiotensin-converting enzyme (ACE) inhibitors or angiotensin receptor blockers (ARBs) are the initial treatment of choice due to their potential to increase nitric oxide release and to reduce bradykinin catabolism, plasminogen activator inhibitor-1 expression, microcirculatory changes, and proteinuria [102, 174-175]. An additional benefit of RAAS inhibitors may be potentiation of the antiangiogenic effects of VEGF-based therapy since angiotensin II–IV (downstream cleavage products of angiotensinogen) upregulates VEGF in tumor tissue [176].

In some individuals, additional antihypertensive medications may be required to control hypertension [175]. If such is the case, non-dihydropyridine calcium channel blockers (e.g., verapamil and diltiazem) should not be used in combination with cytochrome P450 3A4 (CYP3A4) isoenzyme inhibitors (e.g., sorafenib), due to the risk of markedly increased concentrations of the chemotherapeutic agent. If therapy with a calcium-channel blocker is desired, amlodipine or nifedipine are preferred.

If cardiac systolic dysfunction develops, the offending chemotherapeutic agent(s) should be discontinued until the patient has been stabilized and started on appropriate heart failure–based therapy according to guidelines published by the American College of Cardiology, American Heart Association, and Heart Failure Society of America [173]. An ACE inhibitor or ARB, in combination with a beta-blocker, is recommended unless contraindicated. In patients with anthracycline-induced cardiomyopathy, enalapril therapy can reduce the decline in LVEF and subsequent cardiac events [177]. Valsartan, an ARB, blocked the acute cardiotoxic effects of anthracycline treatment during a small randomized trial [178]. Among the beta-blocking agents, carvedilol demonstrated cardioprotection in anthracycline-treated patients, probably by virtue of its intrinsic antioxidant properties [179]. In addition to heart failure therapy, treatment should be instituted for any comorbid conditions that may adversely affect cardiovascular function (i.e., hypertension, diabetes, and hyperlipidemia). Optimal management of hypertension is pivotal to prevent heart failure progression.

Patients with asymptomatic bradycardia or QT interval prolongation as a result of chemotherapy should continue therapy with ongoing monitoring. Conversely, symptomatic patients may require discontinuation of the offending agent (including beta-blockers, calcium channel blockers, and digoxin) and placement of a permanent pacemaker if advanced heart block is present [79]. Multiple myeloma patients who develop symptomatic bradycardia with thalidomide therapy should be considered for pacemaker implantation [180].

Treatment of myocardial ischemia in the setting of chemotherapy may include medical therapy, such as beta blockers, calcium channel blockers, statins, antiplatelet agents, and anticoagulants, and percutaneous coronary intervention (PCI) [181-182]. If PCI is performed, the procedure choice – balloon angioplasty, placement of a bare metal stent, or a drug-eluting stent) -- may be determined by whether the patient can receive dual antiplatelet therapy for an extended period (i.e., six to 12 months, in which case placement of a drug-eluting stent can be considered) or for only a limited time (up to four weeks), in which case a balloon angioplasty or placement of a bare-metal stent is advised.

The decision to administer therapy to prevent VTE events should be individualized based on the patient’s risk factors. Aspirin may be used in selected patients considered to be at increased risk for arterial and VTE complications, especially if platelet count and function are preserved [119]. The International Myeloma Working Group recently issued recommendations regarding the prevention of thalidomide- and lenalidomide-associated thrombosis in myeloma patients [183]. Aspirin (81 to 325 mg) is recommended for low-risk patients, and low-molecular-weight heparin (LMWH), enoxaparin 40 mg, or full-dose Warfarin is recommended for those with two or more risk factors or receiving concomitant high-dose dexamethasone or doxorubicin. The risk factors include age, history of VTE, central venous catheter, comorbidities (infections, diabetes, and cardiac disease), immobilization, surgery, inherited thrombophilia, and hyperviscosity.

Once a VTE is diagnosed, the LMWH (enoxaparin, dalteparin, or nadroparin) should be administered for three to six months, followed by anticoagulant therapy with warfarin or LMWH indefinitely or until cancer remission is achieved [184].

### Radiotherapy

It must be highlighted that radiotherapy itself may be cardiotoxic, so conjunction with chemotherapy that has the same effects may worsen the risk. Nevertheless, further studies need to be done with regard to the combined use of each drug and radiotherapy. This is especially salient in agents like tositumomab, which combine radiation and targeting by monoclonal antibodies in their mechanism of action. It has been demonstrated that radiation of the mediastinum as a result of treatment for early stage breast cancer or Hodgkin’s lymphoma is associated with late cardiac repercussions [185]. For instance, a surgical and autopsy study of radiation-induced heart disease found valve injuries in 70% (12 out of 17 available) of studied hearts, although only eight of these were diagnosed with clinically significant dysfunction. Similarly, 63% of myocardiums (10 out of 16 available) exhibited fibrosis related to radiation [186]. The main mechanisms associated with these phenomena are inflammation and vascular damage, which lead to cellular death [187]. This can be related to heart fibrosis found in the aforementioned study.  

In the case of breast cancer, a case-control study that evaluated women with major coronary events after oncotherapy found that incidental exposure of the heart increased the rate of major coronary events in 7.4% per gray of radiation [188], establishing a direct correlation between the dose of radiotherapy and the risk of developing heart conditions. On the other hand, Hodgkin’s lymphoma patients, in an echography study, were found to present Grade 1+ (scale: 0-3) aortic and/or mitral valvar regurgitation in 24% of the patients (15% aortic, 7% mitral, and 2% both) [189].

### Strain scores in echocardiography

Strain is a term used in echocardiography to describe local shortening, thickening, and lengthening of the myocardium as a measure of regional LV function. It can be measured by tissue Doppler imaging (TDI) or by speckle-tracking echocardiography (STE), the latter being the most widely used strain modality.

Monitoring cardiac function and administration of appropriate therapy during chemotherapy is essential in current clinical practice. Nonetheless, no high-level evidence exists to guide the choice of imaging method or frequency of measurements. LVEF should be measured prior to chemotherapy using echocardiography. Patients who develop heart failure during chemotherapy are treated with standard guideline-based heart failure therapy just as any other heart failure patient [190]. For patients who develop asymptomatic LV dysfunction, however, there is not sufficient evidence to give firm recommendations with regard to medical therapy [191].

When a reduction in LVEF during chemotherapy is established, it may be too late for treatment [192]. Reduction in myocardial strain precedes significant change in LVEF [193]. A relative decrease in global longitudinal strain (GLS) > 15% compared with the baseline is likely to be of clinical significance, whereas a decrease < 8% is not [194]. Although strain imaging may detect subclinical myocardial changes, the value of these changes in predicting a clinical outcome is still unknown. A combination of strain imaging with ultrasensitive troponin has been proposed [191].

## Conclusions

Ongoing efforts are needed to provide a better understanding of the frequency, mechanisms of disease, prevention, and treatment of cardiovascular complications induced by the newer, novel chemotherapeutic agents. Development of a cardio-oncology discipline is warranted, in order to promote task forces aiming at the creation of oncology patient-centered guidelines for the detection, prevention, and treatment of potential cardiovascular side effects associated with newer cancer therapies. 
